# Simultaneous Bilateral Anterior Cruciate Ligament Revision Reconstruction

**DOI:** 10.7759/cureus.41092

**Published:** 2023-06-28

**Authors:** Patrick A Naeger, Paul J Weatherby, Tyler Nsekpong, William Weiss

**Affiliations:** 1 Department of Orthopaedic Surgery and Rehabilitation, University of Texas Medical Branch at Galveston, Galveston, USA; 2 Department of Orthopaedic Surgery, Johns Hopkins University, Baltimore, USA

**Keywords:** bilateral knee arthroscopy, bilateral acl injury, bilateral acl reconstruction, orthopedics sports, revision acl reconstruction, arthroscopic acl reconstruction

## Abstract

There are limited studies in the literature regarding the reconstruction of bilateral anterior cruciate ligament (ACL) injuries in a single-stage setting. However, there have been no published studies describing simultaneous revision reconstructions of previously reconstructed bilateral ACLs. We present the case of a 37-year-old male who underwent previous reconstruction of both ACLs at an outside hospital and presented to our outpatient clinic with instability and pain. Simultaneous bilateral ACL revision reconstruction was performed with the use of tibialis anterior allografts. This case report suggests that single-stage bilateral ACL revision reconstruction is a safe procedure that can provide good results for the patient.

## Introduction

The anterior cruciate ligament (ACL) is a common source of injury with an estimated incidence of 68.6 per 100,000 person-years [1]. Moreover, ligamentous damage can occur through non-contact or contact mechanisms, and physical activities that require continuous pivoting motions increase the likelihood of injury. Reconstruction of the ACL is most often an outpatient procedure conducted mainly through an arthroscopic approach [2]. Bilateral ACL injury is relatively uncommon. According to the literature, athletes who have undergone bilateral ACL reconstruction have a 40% chance of returning to pre-injury levels postoperatively [3]. The success and longevity of an ACL reconstruction depends on a variety of factors such as patient characteristics, surgical technique, and postoperative rehabilitation [4]. When considering the reconstruction of bilateral ACLs, the procedure can either be performed in a single-stage or a double-stage setting. Currently, there is limited literature reporting single-stage bilateral ACL reconstruction. These limited reports suggest that the single-stage procedure is both safe, cost-effective, and has good long-term outcomes [5-9]. However, to our knowledge, there is no reported study that describes a revision procedure of previously reconstructed bilateral ACLs in a single setting. In this report, a case is described in which a single-staged bilateral ACL revision reconstruction is performed.

## Case presentation

We present a case involving a 37-year-old healthy male with a previous history of bilateral ACL reconstructions. He was not overweight and did not have any generalized ligament laxity. The index procedures were done approximately three years prior to presentation to our institution. Both previous ACL reconstruction procedures were performed arthroscopically; however, it was not known what tendon grafts were used originally. The patient presented to our outpatient clinic with a chief complaint of right knee instability and pain. He reported that he previously completed >12 months of postoperative rehabilitation for both knees, but continued to have instability of both knees. He mentioned that his pain was aggravated when descending down stairs or performing pivoting motions. He was unable to recall any recent traumatic injury but stated that his pain was intermittent and worse in the morning. He noted that he exhausted several non-operative treatments including physical therapy and bracing options, which provided marginal relief of pain. 

**Figure 1:**
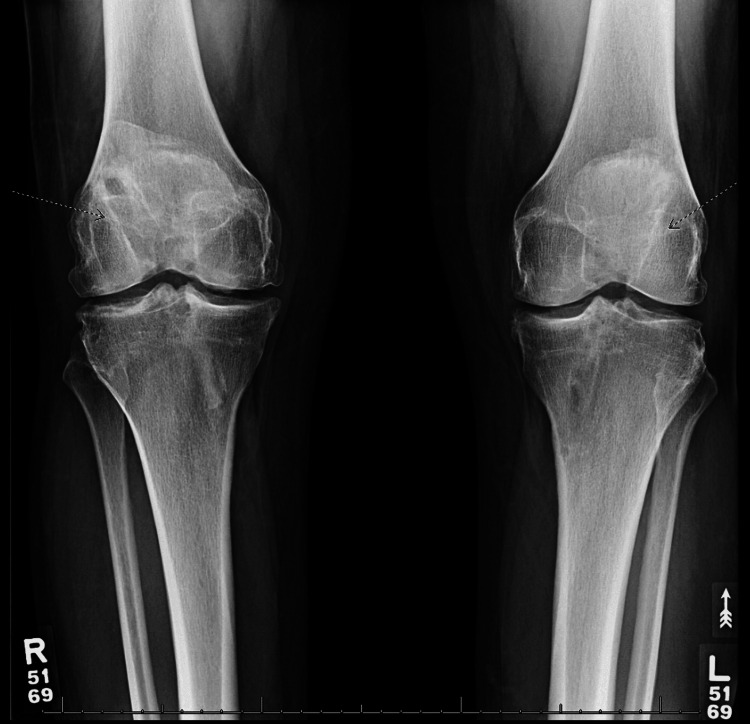
Preoperative radiographs. Bilateral anteroposterior view of the knee showing degenerative changes and vertical femoral tunnel placement (dotted arrows).

**Figure 2:**
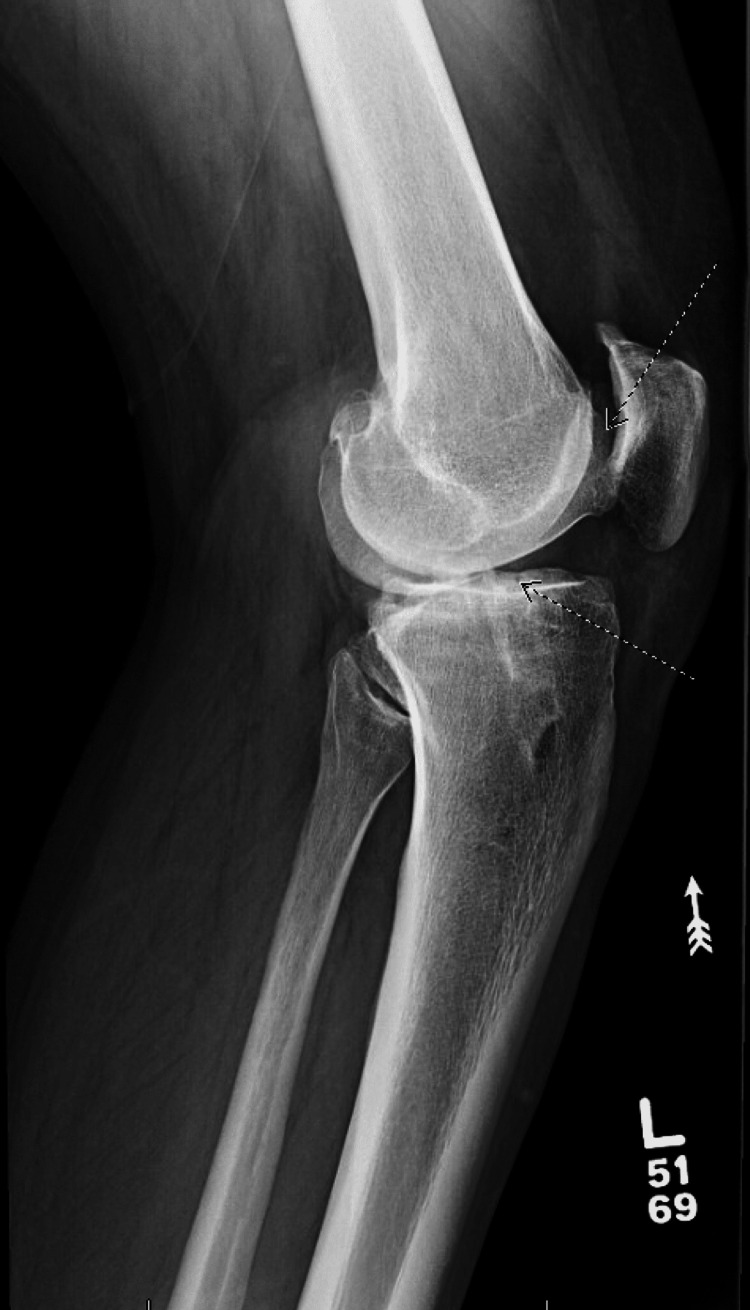
Preoperative radiographs. Lateral view of left knee, showing degenerative changes. Dotted arrows show diffuse joint space narrowing and osteophytes.

**Figure 3:**
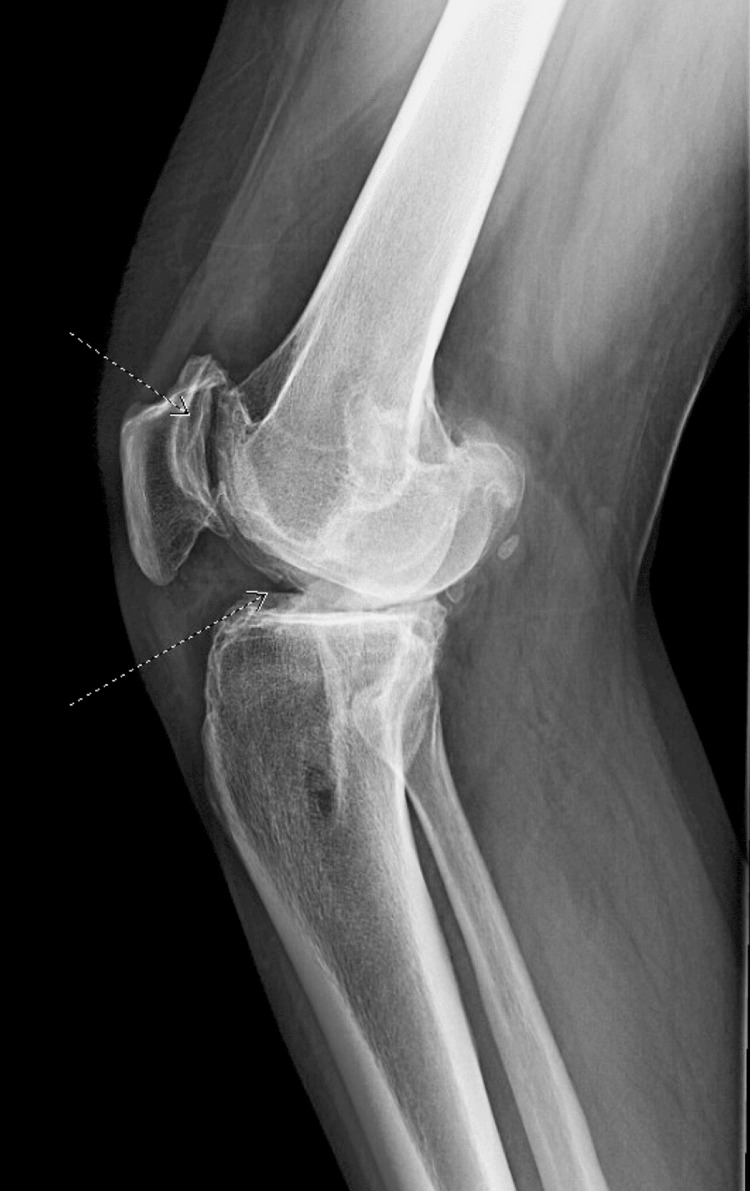
Preoperative radiographs. Lateral view of right knee, showing degenerative changes. Arrows show diffuse joint space narrowing and osteophytes.

**Figure 4:**
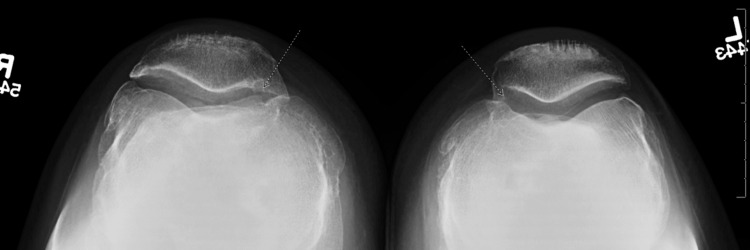
Preoperative radiographs. Bilateral sunrise view of the knees showing degenerative changes. Arrows show bilateral joint space narrowing.

Physical exam revealed a grade IIB Lachmann’s test and positive laxity on the anterior drawer test on the right knee. In addition, the pivot-jerk test was positive and his quadriceps were atrophied upon examination. The left knee demonstrated a grade I Lachmann’s test; however, the patient noted he felt subjectively unstable on the left as well. Bilateral knee X-rays showed vertical tunnel placement from the previous reconstruction procedure and tricompartmental arthritic changes of both knees in which the right was more severe than the left (Figures 1, 2, 3, 4).

**Figure 5:**
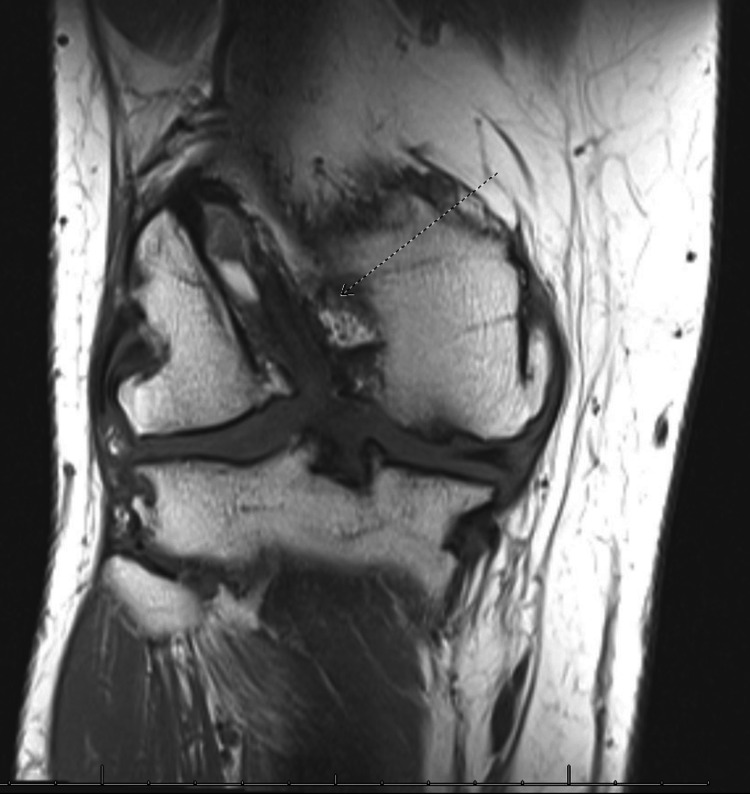
MRI right knee, anteroposterior view showing the previous femoral tunnel (arrow).

**Figure 6:**
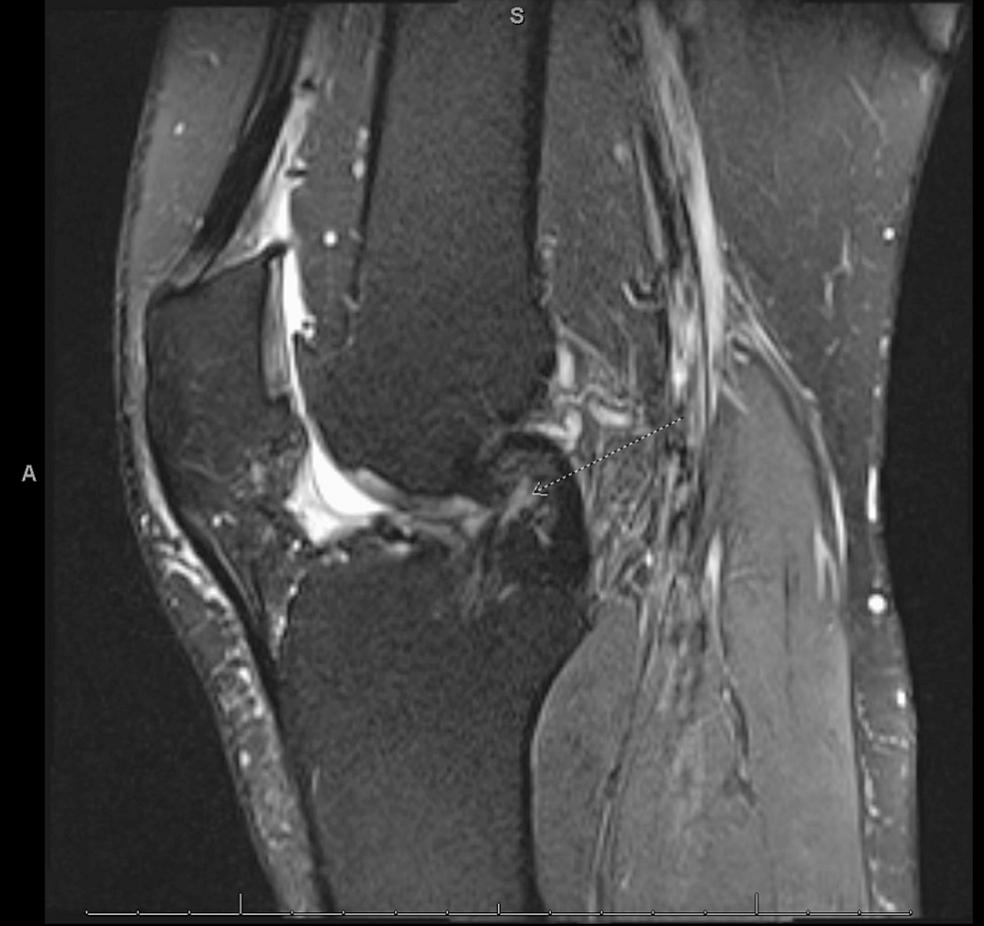
MRI of right knee, lateral view showing previous ACL graft (arrow) with signal changes concerning for a graft tear. ACL: anterior cruciate ligament

**Figure 7:**
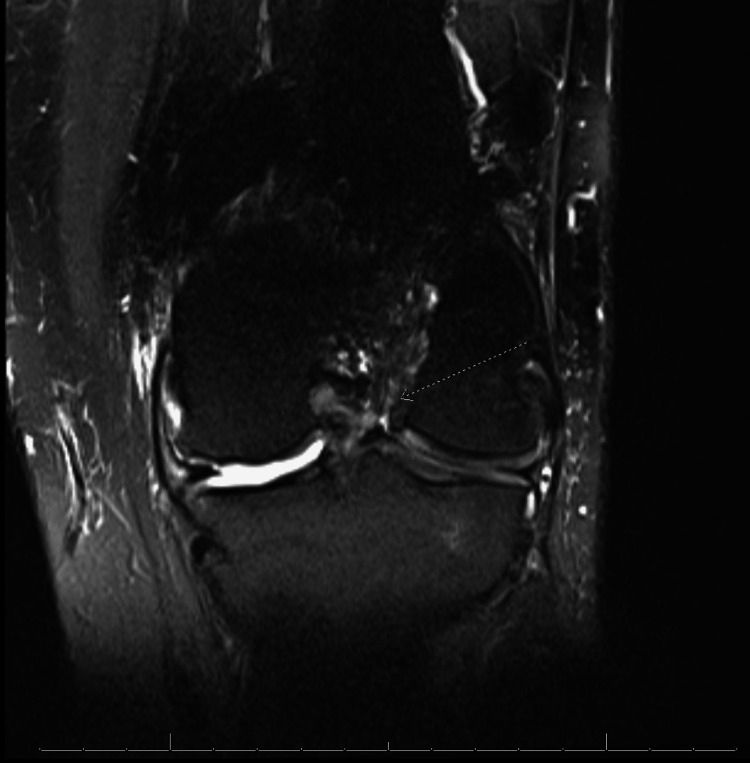
MRI of left knee, anteroposterior view showing previous ACL graft with signal changes concerning for a graft tear (arrow). ACL: anterior cruciate ligament

**Figure 8:**
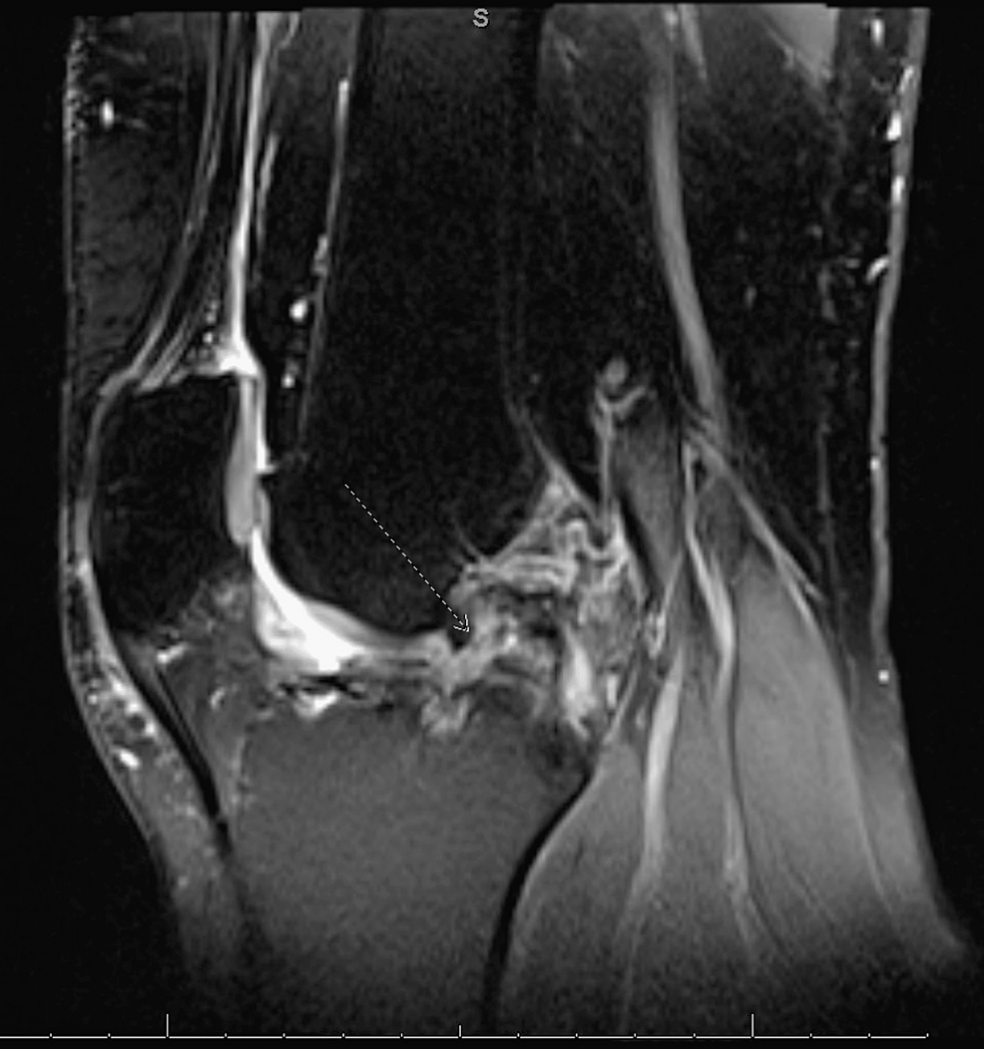
MRI of left knee, lateral view showing previous ACL graft with signal changes concerning for a graft tear (arrow). ACL: anterior cruciate ligament

The decision was made to get advanced imaging in order to evaluate the integrity of the ACL. MRI of the right knee demonstrated evidence of vertical inclination of the previous ACL tunnel in the femur and ossification at the entrance of the femoral tunnel (Figures 5, 6). MRI of the left knee showed evidence of indistinct distal graft fibers, concerning tearing of the previous graft (Figures 7, 8). Since the patient denied any traumatic events, graft failure or improper fixation was the most likely cause for failure. The patient's clinical exam as well as laboratory values did not suggest either knee was infected. Following informed consent and preoperative assessment, the decision was then made to proceed with bilateral, simultaneous ACL revision surgery. 

Preoperative bilateral knee examination was conducted once the patient was asleep and supine, which confirmed previous findings of positive laxity with Lachmann's test and anterior drawer test. The patient was appropriately positioned and draped with sterile precaution to the level of the thigh tourniquet.

An incision was made over the anterolateral portion of the left knee for initial portal placement, followed by medial portal placement, which was made under direct visualization. Diagnostic arthroscopy was then performed starting with the suprapatellar pouch, lateral facet of the patella, lateral gutter, patellofemoral placement articulation/tracking, trochlear groove, medial gutter, and medial joint followed by the lateral joint. For better visualization, a 3.5 mm shaver was used for a limited synovectomy. The medial compartment of the left knee was observed and a previously resected meniscus with degenerative changes was appreciated. Next, the lateral compartment was visualized and mild degenerative changes were observed. 

The ACL and posterior cruciate ligament (PCL) were visualized and assessed. With utilization of the probe, both ligaments were found to be intact, but the ACL exhibited laxity. Debridement of the ACL was conducted followed by widening of the femoral notch. Tibialis anterior grafting was prepared with a 2-0 FiberWire® FiberLoop® (Arthrex, Inc., Naples, Florida, United States) in a whip stitch pattern. The diameter of the graft was sized and measured at 9.0 mm. A tibial tunnel was created using the point-to-point tibial tunnel guide. Arthroscopic visualization was utilized to place the tunnel in the native ACL footprint and was set at 60 degrees. An incision was made at the appropriate location using the guide as a template. The tibial tunnel was drilled using a guidewire and then the tunnel was reamed. A FiberWire was passed through the tibial tunnel for future graft passage.

Next, the femoral tunnel guide was placed at the native ACL insertion of the lateral femoral condyle in the notch under arthroscopic visualization, then a guidewire was drilled through the femur. Bony landmarks such as the intercondylar ridge and bifurcate ridge were used to ensure the anatomic position of the new femoral tunnel. A reamer was used to drill over the guide wire in the femur then the suture was passed through the femoral tunnel exiting the anterior-medial arthroscopic portal. An Endobutton was attached to the graft and then the graft/Endobutton construct was passed through the tibial tunnel into the femoral tunnel. The Endobutton was inverted and secured on the lateral femoral cortex. Tensioning of the knee was conducted through repeated cycles of flexion and extension. An interference screw was placed over the graft in the tibial tunnel with the knee positioned at 30 degrees flexion while pulling tension on the graft. Following placement of the interference screw, a Lachmann’s test was performed for stability, and the ACL was probed for assessment. Lachmann’s test was negative and the ACL was intact. Arthroscopy portals were closed with 3-0 Monocryl (Ethicon, Inc., Raritan, New Jersey, United States), and the tibial incision was closed in layers with 2-0 Vicryl (Ethicon, Inc.) and 3-0 Monocryl. 

The ACL of the right knee was reconstructed in a similar manner. The medial compartment of the knee showed diffuse degenerative changes. In addition, meniscectomy and chondroplasty were performed. Arthroscopic visualization of the ACL and PCL was seen. The PCL was found to be intact. The ACL was ruptured at the femoral insertion and scarred down to the PCL. Anterior tibialis tendon grafting was prepared with 2-0 FiberWire. The graft was sized and measured at 9.5 mm. The tibial tunnel was created with a point-to-point tibial tunnel guide and set at 65 degrees. The Endobutton technique was used to secure the graft similar to the left knee, and an interference screw was placed for fixation. Lachmann’s test was negative and the ACL was found to be intact after assessment. Closing of arthroscopy portals and tibial incision was identical to the left knee. Soft dressings were applied to each leg. 

Postoperatively, the patient was weight bearing as tolerated in a hinged knee brace locked in extension with a range of motion of 0-90 degrees when non-weight bearing. At two weeks follow-up in the outpatient clinic, the patient continued to progress well with full stability of both knee joints and improvement of previous pain. He was able to ambulate without assistance and continued full range-of-motion (ROM) activities with physical therapy. At the six-week follow-up, the patient was continuing ROM exercises with physical therapy and noted decreased pain. On physical exam, the patient had close to full extension of bilateral lower extremities and well-healed surgical incisions. At four months after surgery, our patient demonstrated no pain, full ROM bilaterally, and no instability and subjectively felt better. 

## Discussion

In this case report, we described a 37-year-old healthy male with a history of bilateral ACL reconstruction who underwent a single-staged bilateral ACL revision reconstruction. The main finding in this case report is that single-staged bilateral ACL revision reconstruction can be successful and provide positive outcomes for patients. When looking at the surgical technique for this case, the initial vertical inclination of our patient’s previous femoral tunnel from prior arthroscopic reconstruction did not interfere with our new tunnel placement. This provided the opportunity for a one-stage procedure. Interestingly, there are no reports in the literature of single-stage bilateral ACL reconstructions using hamstring or patellar tendon autograft or allografting. This case is also unique in the fact that we used a tibialis anterior allograft. In addition, the continual progression of our patient postoperatively aligns with previous literature that a single-staged bilateral ACL reconstruction is safe and provides good outcomes [6]. At four months post surgery, our patient demonstrated no pain, full ROM bilaterally, and no instability. He was weight bearing as tolerated without problems and working with physical therapy well. He subjectively felt that both of his knees were more stable. There is also a cost consideration. Single-staged bilateral reconstructions have been reported to decrease financial burden when compared to a two-stage procedure [7] due to having fewer total procedures, insurance considerations, and less opportunity cost of missing work. 

In 2002, Jari and Shelbourne conducted a study regarding bilateral simultaneous ACL reconstruction versus a unilateral operation [5]. The study concluded that there is no significant difference postoperatively between a group of 28 patients who had symptomatic bilateral knee instability from ACL tears [5]. This suggests that a single-staged procedure may be more beneficial for a patient as there will not be additional operation time beyond the first procedure or postoperative recovery resource utilization [5]. In addition, double-staged reconstruction requires more hospital stays, another anesthesia workup, and additional time away from employment [8]. This could not only affect a patient financially but could cause increased physiological stress and increased logistic difficulties. Our case of a bilaterally ACL revision reconstruction also suggests that there is no significant difference postoperatively in patient outcomes than doing two, separate unilateral procedures. There is also no literature currently available for this procedure, which highlights the utility and uniqueness of this case report.

## Conclusions

Simultaneous bilateral ACL revision reconstruction is a reasonable option in select patients and this case report suggests that postoperative outcomes from a single, bilateral ACL revision procedure are no worse than two separate unilateral ACL revision reconstruction procedures. A single simultaneous procedure may also have certain benefits including less anesthesia time, lower economic burden, and fewer logistical considerations. Through this report, we hope to provide support for single-staged procedures, and to serve as a guide for orthopaedic surgeons to use in the case of a bilateral ACL revision reconstruction. 
